# Effects of ultrasonication on increased germination and improved seedling growth of aged grass seeds of tall fescue and Russian wildrye

**DOI:** 10.1038/srep22403

**Published:** 2016-03-01

**Authors:** Juan Liu, Quanzhen Wang, Đura Karagić, Xv Liu, Jian Cui, Jing Gui, Muyu Gu, Wei Gao

**Affiliations:** 1 College of Animal Sci. and Techn., Northwest A&F University, Yangling 712100, Shaanxi Province, China; 2 Institute of Field and Vegetable Crops, Forage Crops Department, Maksima Gorkog 30, 21000 Novi Sad, Serbia; 3 College of Life Science, Northwest A&F University, Yangling 712100, Shaanxi Province, China

## Abstract

The effects of ultrasonic treatments on the germination and seedling growth of aged tall fescue (*Festuca arundinacea*) and Russian wild rye (*Psathyrostaehys juncea* Nevski) seeds were determined using orthogonal matrix experimental design with four ultrasonic factors. The multivariate analysis of variance detected significant differences and coupling effects of the pair-wise factors. The activities of Superoxide Dismutase (SOD) and Peroxidase (POD) and the Malondialdehyde (MDA) content were affected. The ultrasonic treatments had positive effects on the germination percentage (GP) of the aged seeds and the growth of the seedlings (GS) and therefore we provided a basic evidence for the application of ultrasonic treatment to pretreat aged grass seeds. For the four ultrasonic factors, the optimal conditions were a sonication time of 36.7 min, a sonication temperature of 35 °C, an output power of 367 W and a seed soaking time 4.1 h after binary quadratic regressions analyses. The ultrasonic treatment has the potential to improve seedling growth. Moreover, the longevity of the tall fescue and the Russian wild rye seeds was approximately 9.5 and 11.5 years, respectively, under natural conditions of storage. The physiological mechanisms that might contribute to the improved GP and GS were discussed.

Seeds are essential in rebuilding the production capacity of a crop, maintaining the germplasm, and improving species diversity. However, during storage, seeds ageing or deterioration, can significantly leads to a loss of gene diversity, failure to emerge, abnormalities in seedlings, and the eventual reduction of establishment in the field^1^.Studies has showed that the ageing of seeds is associated with serious alterations in the cellular metabolism and biochemistry, including inactivation of enzymes, peroxidation of lipids, disruption of membrane integrity, and damage to Deoxyribonucleic Acid (DNA)^2^. Although the exact mechanisms that cause the ageing of seeds remain elusive, the accumulation of Reactive Oxygen Species (ROS) is considered to be one of the primary causes of seed deterioration^3^.

Generally, dried seeds can be stored for a relatively long period of time, but eventually, the deterioration or ageing of seeds can occur irreversibly^4^. Furthermore, both the prestorage conditions (i.e., temperature and seed moisture content) and genotypic effects play essential roles in affecting the longevity characteristics of seeds^5^. Compared with other pasture plants or crop seeds, the seeds of grasses such as tall fescue (*Festuca arundinacea* Schreb.) and Russian wild rye (*Psathyrostachys juncea* Nevski) have high contents of lipids, which may accelerate the deterioration of the seeds. Thus, extending the longevity and improving the germination ability of stored seeds from both biophysical and economical aspects have become a highly studied problem.

Priming is a pre-sowing treatment that is widely used to promote germination and to improve the quality of seeds under stressed conditions or after ageing from a long period of storage^6^,^7^. The positive effects of priming treatments, which include halopriming, hydropriming, osmopriming, and thermopriming, among others, on seed performance have been demonstrated in many species^8^. However, these treatments are relatively time-consuming with high labour costs^9^. Conversely, the treatment with ultrasound (US), which is easier to operate and is a time saver, generates multiple effects, which include heat and mechanical and chemical effects on seeds, within a short period of time^9^. Additionally, although generally less well tested, US treatments have shown promising results in studies on switch grass^9^ and on many other crops, e.g., *Calanthe* hybrids, beans^10^, corn^11^, barley^12^, fern spores^13^, alfalfa and broccoli^14^, chickpea, wheat, watermelon and sugar delicates^15^,^16^. Therefore, the methods to increase the germination of aged grass seeds and improve seedling growth might be effective; however, no information is available either on the effects of US or the study of thier optimal conditions on the aged seeds of tall fescue and Russian wild rye grasses.

Tall fescue (*Festuca arundinacea* Schreb.) is primarily a cool-season grass species that is found in many pastures and is used for livestock production. It is widely grown throughout the temperate regions of the world^17^,^18^ and is one of the most important forage species that is also used for soil conservation. This species of grass thrives in pastoral environments in which multiple, simultaneous stresses are a common occurrence^19^. Tall fescue (Tf) is also a widely used cold-season turf grass for residential and commercial landscapes^20^, and with superior shade tolerance and a deep green winter colour, this grass has a large advantage over warm-season turf grasses, such as Bermuda grass and zoysia grass.

Russian wild rye (*Psathyrostachys juncea* Nevski) is a perennial grass that grows rapidly, is highly drought and Calcium carbonate(CaCO_3_) tolerant and has a low fertility requirement^21^,^22^,^23^. Russian wild rye is a cool-season forage species that is well adapted to semiarid climates^24^,^25^, and the grass is highly competitive, high-yielding, and an excellent source of forage for livestock and wildlife on semiarid rangelands^26^ in Eurasia and northwest China^22^,^23^,^24^,^27^,^28^,^29^. Therefore, this study examined the effects of US treatments on the attempt to increase the germination of aged seeds and improve the vigour and growth of the seedlings of Tf harvested from the year of 2006, 2010 and 2014 and Russian wild rye grasses (Rw) from 2006 and 2009 respectively. Additionally, by evaluating the alterations of the age-related physiological indicators, we are trying to test whether the US treatment has a positive influence in repairing the damage caused by seed aging process.

## Results

### Germination

**Figure 1:**
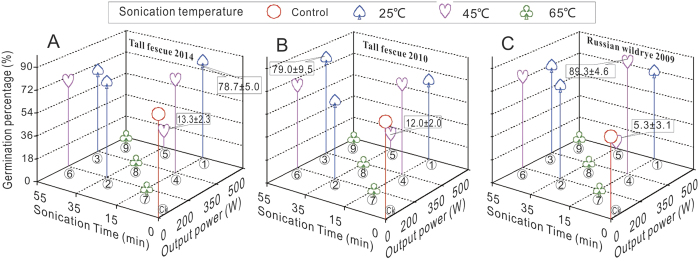
Combined effects of sonication temperature, sonication time and ultrasound output power on the germination of tall fescue (2014 (**A**) and 2010 (**B**)) and Russian wild rye (**C**) seeds. The numbers in the circles indicate the treatments, and Ck is the control. With the exception of treatments 7, 8 and 9, the highest and lowest values are presented.

In groups of Tf harvested at 2006 i.e., Tf (2006), Rw harvested at 2006 and 2003 (Rw 2006 and Rw 2003) of the nested experiment, the seeds did not germinate. Germination also did not occur in treatments 7, 8 and 9 in groups tall fescue harvested at 2014 and 2010 (Tf 2014 and Tf 2010) and Rw (2009) which were at a sonication temperature of 65 °C (Fig. 1A–C). The highes Germination Percentage (GP) was 89.3% in treatment 4 for the Rw(2009) seeds (Fig. 1C), with a sonication time of 15 min, a sonication temperature of 45 °C, an output power of 350 W and a seed soaking time of 1 h. The lowest GP was in treatment 5, and the ultrasonic conditions were a sonication time of 35 min, a sonication temperature of 45 °C, an output power of 500 W and a seed soaking time of 9 h (Fig. 1A–C). At the identical treatment time (15 min) or the identical temperature (45 °C) as that in treatment 4, the highest GP of 78.7% and 79.0% occurred in treatments 1 and 6 for the Tf (2014 and 2010), respectively (Fig. 1A,B). However, the lowest GP among the three groups were also at a temperature of 45 °C in treatment 5 (Fig. 1A–C). The range analyses of the orthogonal design experiments showed that the sonication temperature was the most important factor among the four designed fators.

The GP and Germination Index (GI) were significantly positively correlated with the lengths of the shoots and the roots (Table S1). The lengths of the shoots and the roots were negatively correlated with the Malondialdehyde (MDA) content but were positively correlated with the Superoxide Dismutase (SOD) activity.

The analysis of variance showed that the sonication time, the sonication temperature, the output power and the seed soaking time were significant for the GP, GI, and SVi and the shoot and root lengths (*p* < 0.01), and there were significant coupling effects both pair-wise and among the four factors, with the exceptions of the factors X_3_ and X_4_ and the X_1_*X_2_ interaction for root length (Table 1).

**Figure 2:**
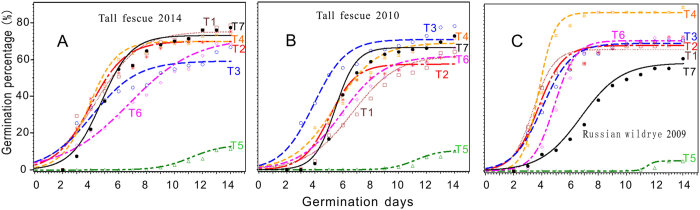
The curves of the models presented germination percentage in the treatments for the tall fescue (2014 (**A**) and 2010 (**B**)) and the Russian wild rye (**C**) seeds. T1 through T6 are the treatments, and T7 is the control (in black). All of the models were significant at *P* < 0.05.

In group Rw (2009), the germination of seeds in all treatments was significantly higher than that in the control (Fig. 2C). The sonication treatments clearly and consistently increased the GP of the Russian wild rye seeds from 2009 (Fig. 2C). The lowest GP was in treatment 5 (T5) among the three groups (Fig. 2A–C), whereas the highest GP was in treatment 4 (T4) for the Rw seeds from 2009 (Fig. 2C). For the new (2014, Fig. 2A) tall fescue seeds, the GP of the seeds in treatment 1(T1) was higher than that in the control (T7), and the GP in treatment 3 (T3) was lower than that in the control (Fig. 2A); these results were in contrast with those for the GP of the old seeds (2010, Fig. 2B). The basic statistic of the permination percentages were plotted in Fig. S1.

### Physiological responses

**Figure 3:**
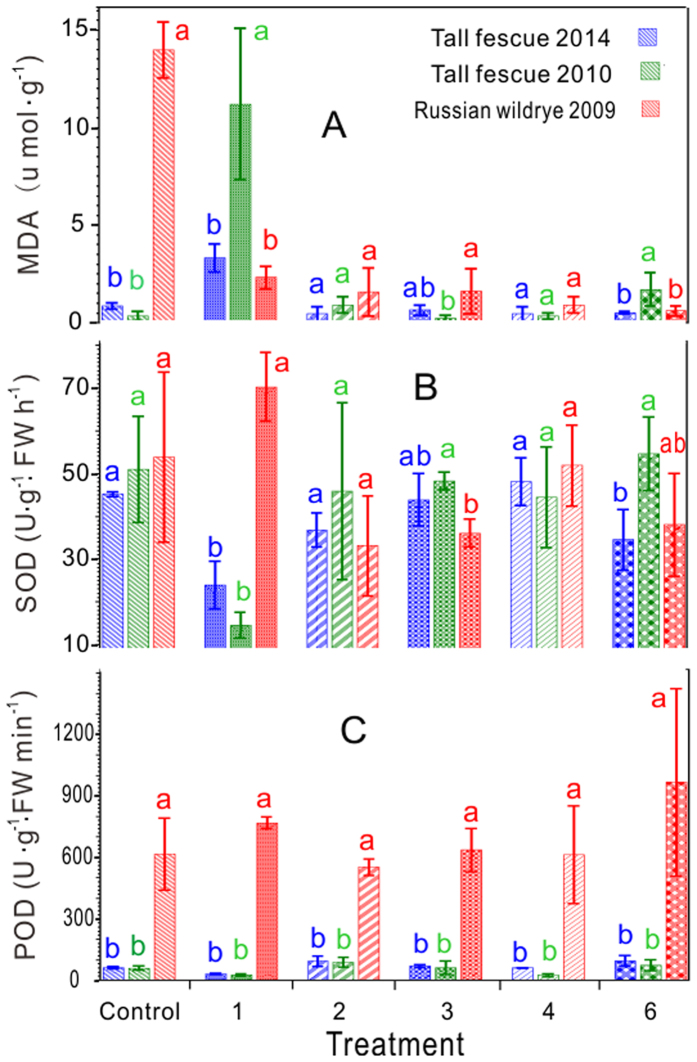
MDA content (**A**) and activity of SOD (**B**) and POD (**C**) in the seedlings of tall fescue (2014 and 2010) and Russian wild rye (2009). The letters indicate differences only within the treatments among the different years. FW is the fresh weight.

In the controls, the MDA content and the activity of POD in the Rw(2009) seedlings from were significantly higher than those of the Tf seedlings from 2014 or 2010 (Fig. 3A,C). There were significantly highest activities of POD in all of the treatments in Rw (2009)(Fig. 3C). In the group of Tf (2010) , in treatment 1, the MDA content was the highest significantly and the SOD activity was the lowest, but the opposite results were found for treatment 3 (Fig. 3A,B). Furthermore, the trends in the MDA content and the activity of SOD were opposite to the changes observed for the group of Rw seedlings from 2009 and those of the Tf (2010) in treatment 1 (Fig. 3A,B). Based on the analysis of variance, the effects of the four factors were significant for the MDA content and for the SOD and POD activities (*p* < 0.01), and there were significant coupling effects both pair-wise and among the four factors, with the exception of factors X_2_ and X_4_ and interaction X_3_*X_4_ on the activity of POD (Table 2).

The multivariate analysis of variance detected significant differences among the factors at *p* < 0.0001 (Table 3).

### Seedling growth

**Figure 4:**
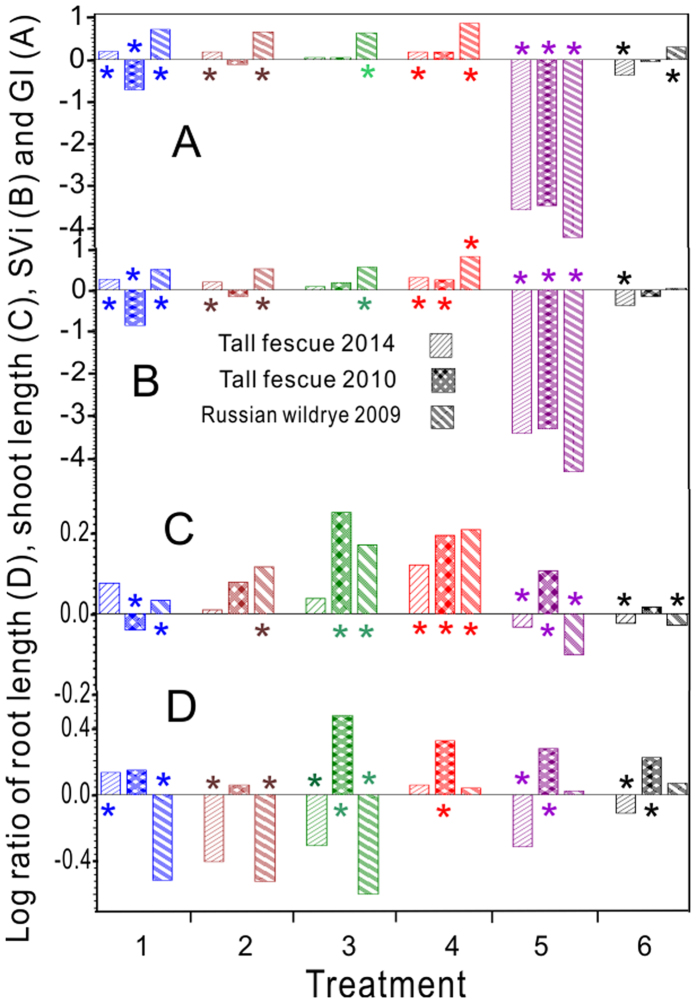
Log ratios of germination index (GI, **A**), seed vigour index (SVi, **B**), and lengths of shoots (**C**) and roots (**D**). * Indicates the difference between the treatment and the control was significant at *p* < 0.05.

In treatment 1, the log ratios of the GI, SVi and shoot length in the group of Rw seedlings from 2009 were significantly higher than those in the controls, whereas these values for the Tf (2010) were lower than those of the controls (Fig. 4A–C). In the Rw (2009), the lengths of the shoots were significantly higher and the lengths of the roots were lower than those of the control in treatments 1, 2 and 3 (Fig. 4C,D). In treatment 5, the lengths of both the shoots and the roots were significantly higher than those in the control in the group of the Tf (2010), but in the group from 2014, the results were the opposite (Fig. 4C,D). The Gis and SVis in the three groups of years were significantly lower than those values in the controls (Fig. 4A,B).

### Optimizing

**Figure 5:**
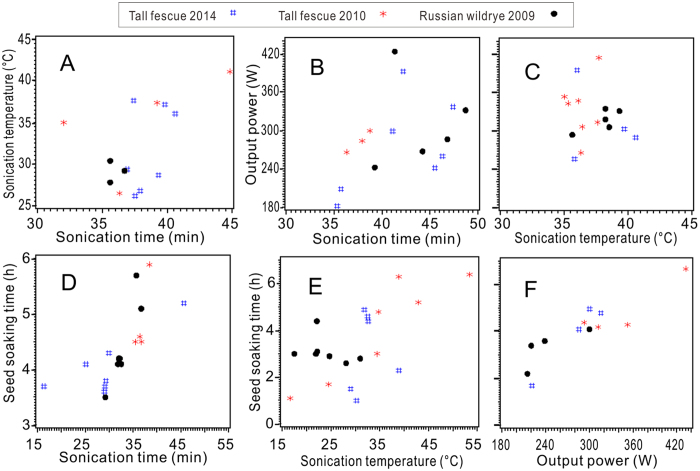
Scatter plots show the stationary points of the optimal models related to sonication temperature with sonication time (**A**), output power with sonication time (**B**) and with sonication temperature (**C**) and seed soaking time with sonication time (**D**), sonication temperature (**E**) and output power (**F**).

A total of 112 quadratic models were selected from the variables of length of shoot, length of root, ratio of shoot/root, length of seedling, GP, Gi and SVi, which were regressed models via pair wise factors of the sonications. The critical values of the stationary points were plotted pair-wise (Fig. 5A through F). The sample sizes of the critical values of the four factors were 59, 57, 51 and 56, which were determined from the model analyses. The mode of the values of the four factors was calculated and was used as the optimum. Thus, the optimal values of the four factors were a sonication time at 36.7 min, a sonication temperature at 35 °C, an output power of 367 W and a seed soaking time of 4.1 h.

## Discussion

Ultrasound technology (US) has been widely applied in medicine , biology^30^and the enhancement of food technological properties (e.g. emulsification ability, solubility and texture) as well as on applications such as homogenization, extraction, viscosity alteration, crystallization, drying, and defoaming^31^,^32^,^33^,^34^. By the combiniton of treatment of pressure or heat, the synergistic effect of US can be very efficient on inactivition of microorganisms and enzymes such as peroxidase and lipooxygenase in the case of food storage^34^. To explain the roles of US treatments in seed priming, many biochemical and physiological mechanisms have been suggested, which include the renovation of the age-related cellular damage and an acceleration of the metabolic imbibitions that activate the protrusion of the radicles caused by the effects of cavitational activity^12^. In this study, we investigated the effects of different levels of output power, times, and temperatures of ultrasound treatment on Tf and Rw seeds to determine the optimal conditions promoting germination and seedling growth. The GP and the lengths of roots and shoots in all treatments were significantly higher than those in the control, particularly for the seeds stored a long time (for GP:79.0% v.s 54% in Tf,2010; and 89.3% v.s. 36% in Rw,2009)(Fig. 1B,C); this result was probably because the ultrasound treatment increases the porosity of the seeds by acoustic cavitation facilitating oxygen availability and water uptake^35^, which are the necessities for seeds to initiate the first step of germination. And some other interesting studies showed that ultrasonic waves can efficiently accelerate the starch metabolism by activating the enzyme (alpha-amylase) occurs during seed germination^12^,^36^. Similarly, the germination of barley seeds with ultrasonic treatment increased approximately 1.042- to 1.065-fold relative to the controls, and the germination period was significantly reduced by 30–45% compared with controls^12^. The results of the present study for the Rw (2009) seeds (Fig. 2C) were consistent with that study. And for seedling growth values described as (Gi, Svi, lenghes of roots and shoots) in treatment 4 of the two species were considerably greater than the control group (Fig. 4A–D). Additionally, although the germination models were significant at *p* < 0.05, the comparably variations indicated the effects of US treatments^9^ (Fig. 2, Table S2 and eq. 3). The physiological role of the US treatment may be involved in acting a catalytical base point in the enhancement of seedling performances, especially for those natrual aged seeds whose protective enzyme systems were considerably damaged by reactive oxygen species (ROS).

The optimization of the conditions in the multifactor interaction process using an orthogonal design was effective and reliable^9^,^37^. Based on the results of the present study, the optimization of the four factors resulted in a sonication time of 36.7 min, a sonication temperature of 35 °C, an output power of 367 W and a seed soaking time of 4.1 h. For the critical values, the factor sample sizes were 59, 57, 51 and 56, respectively, which were treated as large sample statistics (n > 30) because of the diverse results under complex effects 38,39. The collapse of cavitation bubbles produced by a series of compression and rarefaction during the ultrasonic process in aqueous medium generates shear forces that can induce mechanical and chemical effects on seeds immersed^34^. Under the optimal conditions of US treatment in this study, aged seeds can be stimulated appropriately and were prepared, in the state of physicochemical, well enough to initiate germination and seedling emergence . A wide range of optimal conditions was determined for the ultrasound treatment for various types of plants: 60 W, 22 °C, and 2 min for spruce^40^; 460 W, 30 °C, and 15 min for barley 12; 135 W and less than 7 min for *Calanthe* hybrids 15; and 45 min for chickpeas^16^. The reasons for these distinctions might be associated with the different types of species or the individual seed characteristics (e.g., thickness of seed coat, size, infectious microbes and dormancy). With appropriate ultrasound conditions relatively easy to cooperate and lowcost, the damage caused by aging of seeds (especailly of those rare plant species) could be minimized. Second, the total energy balance principle might explain the various results in ultrasonic performances. Compared with a previous study^37^, a lower sonication temperature (35 °Cvs. 39.7 °C in the previous) , output power (267 W vs. 348 W) and a longer sonication time (36.7 min vs. 22.5 min) and seed soaking time (4.1 h vs. 0 h) were required. Additionally, the ultrasonic treatment was successful in this experiment, i.e., for the GPs of the Tf (2010) and of the Rw (2009) (Fig. 1B,C); the highest GP value was at 55 min of sonication time and 200 W of output power in the former group (Fig. 1B) and was 15 min and 350 W in the latter group (Fig. 1C). Moreover, The germination capacity and seedling growth in aged seeds in our study both increased with US treatment, while no significant improved germinations were observed on non-aged seeds treated although shown influences in the models (eq. 3, Table S2 and Fig. 2), which could be suggested that the intracellular redistribution of water^3^, damaged of embryo cell ultrasytucture^5^, shifts in cellular pH and redox state^7^ involved in natrual aging were relatively repaired by the effects of US priming treatment.

Interestingly, the critical lethal temperature of ultrasonic treatment occurred at or below 65 °C, which was demonstrated in treatments 7, 8 and 9 (Fig. 1A–C). Overall, with an increase in sonication time, sonication temperature or ultrasound output power, physical and chemical damages occurs due to the very rapid localized changes in pressure and temperature induced by ultrasound wave causing shear disruption, intensive cavitation, thinning of cell membranes,localized heating, and free radical production^34^, which can produce lethal effects on seeds immersed in the bath. US treatments longer than 5 min had a negative effect on the germination rates of pepper seeds^16^. For ginger particle surfaces when ultrasound was used, a heavily damaged region was observed within 200 min^41^. In addition, some reactions such as protein denaturation, molecular degradation, and starch gelatinization can occur during processing at high temperature^42^ (65 °C possibly in the study), which may significantly damage seeds vigor during the hydration process. Besides, asymmetric implosions of the cavitation bubbles close to seeds surface are likely to produce microjets that can affect mass transfer. More complex mechanisms to explain the damage induced by US treatment require further investigation because of the significant coupling effects among the factors (Tables 1, 2, 3).

The accumulation of reactive oxygen species (ROS) is considered to be the primary factor that leads to the ageing of seeds during storage^43^. The proteins and nucleic acids of mature seeds are damaged by excessive ROS, which may accelerate reactions on peroxidation of lipids accelerating disintegration of membranes^2^,^44^. The MDA is the end product of lipid peroxidation , accumulating gradually in seeds as the seeds deteriorate during storage^2^, which was consistent with the results of the present study. In the group of Rw (2009), the MDA contents in all treatments (treatments 1–4 and 6) were significantly decreased compared with the control (Fig. 3A), which suggested that the recovery of membrane integrity was effective in the treated aged seeds. The great decrease also may largely because a certain intensity of ultrasound wave performed can efficiently inactivite enzymes like lipooxygenase of aged seeds. And for food storage, studies have showed that the use of the combination of heat and ultrasound was successful in inactivting enzymes such as peroxidase and lipooxygenase^34^. Accordingly, the cellular protective ability scavenging the accumulation of ROS induced aging of the seeds was evaluated. The SOD activity directly modulates the amount of ROS^37^. In treatments 1 and 3 of the present study, complementary trends were observed for MDA and SOD, both in the Tf (2010) and in the Rw (2009) (Fig. 3A,B); an identical results for GP and SVi was observed in the two groups of grasses (Fig. 4A,B). However, the trend changed and was the opposite for the lengths of the shoots and the roots (Fig. 4C,D). The changes in the SOD activity under abiotic stress might reflect the identical trend of the changes in the superoxide radical production^37^, which were likely a result of electron leakage from the electron transport chains to molecular oxygen^45^. The increase in the SOD activity was possibly correlated with both the temporal regulation of specific isoenzymes and the induction of new isoforms^46^. Further research is required on the SOD isoenzymes with regard to the grasses under ultrasonic treatment. The antioxidant enzymes such as POD in plant cells remove the active oxygen caused by plant stress^47^ and therefore effectively prevent its accumulation . The destruction of these antioxidant enzymes or the reduction of their activities would accelerate seed ageing. In the present study, the POD activity in the group of Rw (2009) was significantly higher than that in the other two older groups (Fig. 3C). Similarly, the optimal use of ultrasound-assisted extraction (UAE) for the extraction of phenolic compounds from *Cratoxylum formosum* leaves resulted in a higher efficiency of strong antioxidant activities to protect H_2_O_2_ induced cell death^48^. The difference might be partially explained because of the different plant species. The lengths of the shoots and the roots and the Gi and SVi values were significantly different from those of the controls in the three groups (Fig. 5A–D), which was consistent with the previous study^9^. The lengths were large sample statistics (n = 30)^38^,^39^, and the ultrasonic treatments significantly affected the GP and the seedling growth.

Additionally, in present study, no germination or seedling growth performances in any of nine treatments during the experiments occurred for either the treated groups or the controls of the grass seeds (the tall fescue from 2006 and the Russian wild rye from 2006 and 2003), which indicated that the longevity of the tall fescue and the Russian wild rye seeds was approximately 9–10 years and 10–12 years, respectively, in natural storage conditions (room temperature, with an approximately 13% moisture content). The longevity of seeds depends not only on the genetic characteristics of the seed but also on the physiological state, seed moisture content, harvest temperature, and storage methods, among others^49^. The classification described by Ewart^50^ showed that the longevity for most types of grass seeds stored under natural conditions was 3 to 15 years because after the completion of dormancy in a very short period of time, the seeds of grass soon lose the ability to germinate^51^. Furthermore, the GP of wheatgrass (*Agropyron cristatum*) was 33.0% when stored for 8 years, which then declined to zero in the 9^th^ year. Similarly, when stored for 9 years, the GP of *Bromus inermis* and Siberian wild rye (*Elymus sibiricus*) was 45% and 64%, which then decreased to zero in the 11^th^ and the 10^th^ year, respectively^52^ . All the evidences above further supported suggestions that the life span of most grass seeds is approximately 10 years in natural conditions.

Based on the orthogonal experimental design, which is balanced, separable or not mixed^53^, eight variables of seedling growth and germination composed 112 binary quadratic models with pair wise independent variable among the designed factors. Therefore, the results of the optimizing was mathematically reliable due to using the big sample of 223 critical values of the stationary points derived from the 112 models (Fig. 5) and the mode calculated^38^,^39^.

## Conclusions

The ultrasonic treatment had positive effects on the germination of aged seeds and seedling growth, which provided the basic evidence for the further application of ultrasonic treatment to pretreat aged grass seeds. The optimal conditions of the four factors were a sonication time of 36.7 min, a sonication temperature of 35 °C, an output power of 367 W and a seed soaking time for 4.1 h. Additionally, the method is simple, cheap and a time saver, and the ultrasonic treatment has the potential for use to improve seedling growth. Moreover, in the present study, the longevity of the tall fescue and Russian wild rye seeds was demonstrated indirectly to be approximately 9.5 and 11.5 years under conditions of natural storage; however, the determination of the exact duration of the natural ageing process requires more insightful explorations in the future.

## Methods

### Seed materials and experimental design

Tall fescue (*Festuca arundinacea* Schreb.) and Russian wild rye (*Psathyrostachys juncea* Nevski) seeds were obtained from the China Agricultural University Grassland Research Station located at the Hexi Corridor in Jiuquan, Gansu Province, China. This study was performed at the Laboratory of the Grassland Science Department, Northwest A&F University, Shaanxi Province, China. The seeds of the grasses were stored for 1 to 12 years at room temperature in the laboratory. Six groups of the seeds of the two grasses composed the nested experiments (Table S3). Each group was fixed in an orthogonal matrix design [L_9_ (3^4^)] for ultrasonic treatment.

Four factors were examined, namely, the sonication time (factor A), the sonication temperature (B), the ultrasound output power (C) and the seed soaking time (D). Based on the orthogonal design, each of the four ultrasonic irradiation factors was assigned three levels (Table 4), and nine treatment combinations for the different factors were established^53^. Additionally, a control was included without ultrasonic and soaking treatment (Table S4). All of the treatments were performed in triplicate.

Ultrasonic irradiation was produced with an ultrasound generator (KQ-500DE; Kunshan Ultrasound Instrument Co., Ltd., China) with a fixed 40 kHz frequency, an adjustable temperature (10 to 80 °C) and ultrasonic power that ranged from 200 to 500 W. It consisted of a stainless steel vessel (22.5 L capacity), connected to a piezoelectric element, having water inlet and outlet valves. The water in ultrosonic bath which is circulating had been heated to the required temperature level before the process and the water was insulated in order to minimize the heat transfer through the surroundings. Three levels of volumetric power (0.013w/ml; 0.029w/ml and 0.033w/ml) determined^33^ were respectively fixed in the corresponding experimental conditions set (ultrsonic time, temperature, out power). During the experiment, each group of seeds (soaked previously) which had been coated with gauze (based on the orthogonal design [L_9_ (3^4^)]) were immersed into the distilled water (13 L, requried temperature) in the bath and then the equipment was turned on for corresponding conditions (showed as Table 4 and Table S4) respectively, while the water temperature was monitored and needs to be intermittently checked to ensure that the temperature remained constant during each treatment.

Additionally, an electrothermal constant-temperature oven (DHG-9140A; Shanghai Yiheng Instrument Co., Ltd., China), a plant incubator (ZPW-400; Harbin DongTou SG-Tech Development Co., Ltd., China) and an electronic analytical balance (YP1200; Shanghai Science and Industrial Co., Ltd., China) were used.

### Germination Tests

The seeds were surface sterilized in a 0.1% (w/v) sodium hypochlorite solution for 15 min, rinsed five times with distilled water, and germinated in 100-mm sterile Petri dishes lined with two layers of Whatman No.1 filter paper that were moistened with distilled water or with the treatment solutions. The germination tests were repeated three times with 50 seeds per treatment. The Petri dishes were wrapped with transparent plastic wrap to prevent the evaporation of water. The water level in germinator reservoir was maintained by inputs of distilled water daily. The Petri dishes were placed in a germinator (LRH-250-GS II; China) set to an alternating diurnal regime of 16 h of light with 5500 Lx at 25 °C and 8 h of dark at 20 °C for 14 days, which was evaluated according to the study made by Lu^54^. This temperature regime was chosen to represent mid-spring temperatures, which corresponded to the time of year when the seeds of the grasses germinated^54^. The germinated seeds were counted each day for 14 days. The shoot and root lengths were measured on the fourteenth day after sowing, and 10 seedlings were measured from each Petri dish of the three repetitions. The sample size was 30 (3 × 10). When the total number of seedlings was fewer than 10, all seedlings were measured by rules from ISTA (International Seed Testing Association). The germination of a seed was defined as the elongation of the coleoptiles to 0.2 cm. The germination percentage (GP) was calculated. The germination index (Gi) was estimated using the following formula:


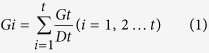


where *Gt* is the number of germinated seeds within a day, *Dt* is the corresponding number of germination days and t is the number of total germination period (14 days).

The seed vigour index (SVi) was determined using the following formula:


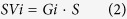


where S is length of shoot.

### Physiological parameter determinations

The activity of SOD and POD and the MDA content of the treatments were determined. The SOD activity was determined according to the method of Zhang^55^. For the antioxidant enzymes extractions, each group of seedlings (0.5g) were homogenized in 5 ml 50mM sodium phosphate buffer (PH 7.8 for SOD and POD containing 1% (w/v) PVP and 0.1 mM Na_2_EDTA.) The homogenate then was filtered with two layers of filter paper and centrifuged at 10000 g for 15 min at 4  °C, after which, aliquots of the supernatant were used to determine the enzyme activities(SOD and POD) at 25 °C. The SOD activity was measured spectrophotometrically as described by Beyer and Fridovich^56^ and one unit of SOD activity was defined as the amount of enzyme that inhibited 50% of NBT photoreduction^57^. POD activity was measured using guaiacol (1-hydroxy-2-methoxybenzene, C_7_H_8_O_2_) as a substrate by the method described by Fu. The reaction mixture contained 50 μl of 20 mM guaiacol, 2.8 ml of 10 mM phosphate buffer (pH 7.8), and 0.1 ml enzyme extract. The reaction was started with 20 μl of 40 mM H_2_O_2_.One unit of POD activity was defined as the amount of enzyme that increased the optical density by one absorbance unit at 470 nm per minute^56^.. The malondialdehyde (MDA) content was determined using the thiobarbituric acid (TBA) reaction, according to the method of Madhava and Sresty^58^. For measurement of MDA content, each group of sample containing 0.1 g homogenate of seedlings was mixed with 5 ml TCA (0.5%) and centrifuged at 10,000 × *g* for 25 min. The mixture then was heated at 95 °C for 30 min and then an ice bath was used to cool the tubes quickly. After the tube was centrifuged at 10 000 × *g* for 10 min, the absorbancy of supernatant was used for the determination of the MDA content. The value for the nonspecific absorption at *A*_600_ was subtracted from the *A*_532_ reading. The concentration of MDA was calculated using MDA’s extinction coefficient of 155 mM^−1^ cm^−1^ 57.

### Data analyses and statistical methods

The data were subjected to analysis of variance (ANOVA) using the SAS statistical software package (version 8.2)^59^. Differences between the means were tested with Student-Newman-Keuls tests, and values of p < 0.05 were significantly different.^38^,^39^,^60^

The germination percentages were simulated using the following logistic model^38^,^39^,^60^


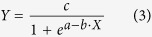


where *c*, *a* and *b* are constants (Table S2), X is germination days. The models were significant at *P*r < 0.05. The curves of the models were presented in Fig. 2 and the basic data were listed in Table S5.

For the generic results, the variables (factors A, B, C and D) were denoted as X_1_ to X_4_. The dependent variables, namely, GP, Gi, SVi, length of shoot, length of root, ratio of shoot/root, length of seedling (shoot + root), content of MDA, and activities of SOD and POD, were denoted as Y_1_ to Y_10_, respectively. These variables of the experimental group were individually approached and analysed via pair-wise, variable (X_1_ and X_2_, X_1_ and X_3_, X_2_ and X_3_, X_1_ and X_4_, X_2_ and X_4_, and X_3_ and X_4_) quadratic regression models^38^,^39^,^59^


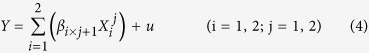


where β is a constant. The critical values of the stationary points (X_1_, X_2_, X_3_ and X_4_) were obtained from the significant quadratic models. The thickest value was used as the mode^39^. The mode of the values was calculated using the following formulas:


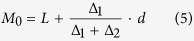



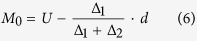


where L is the lower limit value of the array in which the mode was located, U is the upper limit value of the array in which the mode was located, ∆_1_ is the distance of the frequency between the lower adjacent array and the mode array, ∆_2_ is the distance of the frequency between the upper adjacent array and the mode array, and *d* is the distance between the arrays. These analyses and graphical procedures were performed using the SAS statistical software package (v8.2)^59^.

## Additional Information

**How to cite this article**: Liu, J. *et al.* Effects of ultrasonication on increased germination and improved seedling growth of aged grass seeds of tall fescue and Russian wildrye. *Sci. Rep.* **6**, 22403; doi: 10.1038/srep22403 (2016).

## Supplementary Material

Supplementary Information

Supplementary Dataset

## Figures and Tables

**Table 1:** Analyses of variance for the model of germination percentage, germination index, seed vigour index, shoot length and root length for each of the experimental factors and the interactions among them.

Source (Factors)	DF	Germination percentage (%)		Germination index		Seed vigour index		Shoot length (cm)		Root length (cm)	
		*F*-value	*P*r>*F*	*F*-value	*P*r>*F*	*F*-value	*P*r>*F*	*F*-value	*P*r>*F*	*F*-value	*P*r>*F*
X_1_	3	44.31	<.0001	15.93	<.0001	13.04	<.0001	3.97	0.0081	5.52	0.0010
X_2_	2	25.89	<.0001	16.03	<.0001	11.99	<.0001	3.74	0.0243	13.70	<.0001
X_3_	3	42.16	<.0001	19.50	<.0001	17.56	<.0001	13.84	<.0001	1.66	**0.1786**
X_4_	3	40.46	<.0001	16.86	<.0001	13.32	<.0001	7.68	<.0001	1.27	**0.2832**
X_1_*X_2_	1	109.58	<.0001	53.50	<.0001	43.53	<.0001	28.15	<.0001	0.10	**0.7579**
X_1_*X_3_	3	53.79	<.0001	28.79	<.0001	22.50	<.0001	11.88	<.0001	4.16	0.0025
X_2_*X_3_	1	116.02	<.0001	48.59	<.0001	29.97	<.0001	10.01	<.0001	9.17	<.0001
X_1_*X_4_	3	53.79	<.0001	28.79	<.0001	22.50	<.0001	11.88	<.0001	9.17	<.0001
X_2_*X_4_	1	121.13	<.0001	51.49	<.0001	42.68	<.0001	17.00	<.0001	12.84	0.0004
X_3_*X_4_	3	55.94	<.0001	36.49	<.0001	17.99	<.0001	2.00	0.1126	13.02	<.0001
Model	62	16.71	<.0001	9.23	<.0001	8.01	<.0001	6.13	<.0001	9.20	<.0001
R^2^		0.882		0.815		0.792		0.167		0.232	

The factors X_1_, X_2_, X_3_ and X_4_ represent sonication time, sonication temperature, output power and seed soaking time, respectively.

**Table 2:** Analyses of variance for the model of MDA, SOD and POD for each of the experimental factors and the interactions among them.

Source (Factors)	DF	MDA		SOD		POD	
		*F*Value	*P*r>*F*	*F* Value	*P*r>*F*	*F* Value	*P*r>*F*
X_1_	3	19.09	<.0001	6.69	0.0015	12.13	<.0001
X_2_	2	10.84	0.0003	5.81	0.0078	0.44	**0.6455**
X_3_	3	48.58	<.0001	14.13	<.0001	19.49	<.0001
X_4_	3	26.08	<.0001	6.41	0.0019	1.23	**0.3181**
X_1_*X_2_	1	109.64	<.0001	21.58	<.0001	29.12	<.0001
X_1_*X_3_	3	43.77	<.0001	11.06	<.0001	10.01	0.0001
X_2_*X_3_	1	41.73	<.0001	10.41	<.0001	16.98	<.0001
X_1_*X_4_	3	48.77	<.0001	11.07	<.0001	10.00	0.0001
X_2_*X_4_	1	88.67	<.0001	22.48	<.0001	61.82	<.0001
X_3_*X_4_	3	14.29	<.0001	3.62	0.0252	2.70	**0.0646**
Model	62	20.77	<.0001	5.16	0.0001	5.93	<.0001
R^2^		0.906		0.706		0.733	

The factors X_1_, X_2_, X_3_ and X_4_ represent sonication time, sonication temperature, output power and seed soaking time, respectively.

**Table 3:** Multivariate Analysis of Variance - MANOVA Test Criteria and exact F-statistics for the Hypothesis of No Overall Effects of the factors.

Factors	Statistic	Wilks’ Lambda	Pillai’s Trace	Hotelling-Lawley Trace	Roy’s Greatest Root
Sonication time	Value	0.3371	0.6629	1.9668	1.9668
	*P*r > *F*	<.0001	<.0001	<.0001	<.0001
Sonication temperature	Value	0.5730	0.4270	0.7452	0.7452
	*P*r > *F*	<.0001	<.0001	<.0001	<.0001
Output power	Value	0.3279	0.6721	2.0494	2.0494
	*P*r > *F*	<.0001	<.0001	<.0001	<.0001
Seed soaking time	Value	0.3371	0.6629	1.9668	1.9668
	*P*r > *F*	<.0001	<.0001	<.0001	<.0001

H = Type III SSCP Matrix for sonication time, sonication temperature, output power and seed soaking time.

**Table 4:** Assignment of control factors and levels in the nested experimental design using an orthogonal matrix (L_9_ (3^4^)).

Factors	Sonication time (min)	Sonication temperature ( °C)	Output power (W)	Seed soaking time (h)
Level 1	15	25	200	1
Level 2	35	45	350	5
Level 3	55	65	500	9
