# Supplementary material for: Effects of ultrasonication on increased germination and improved seedling growth of aged grass seeds of tall fescue and Russian wildrye
Source: Sci Rep. 2016 Mar 1;6:22403. doi: 10.1038/srep22403 (PMC4772161; doi:10.1038/srep22403)
Supplement: Supplementary Information [file srep22403-s1.pdf]

Supplementary Information:

Manuscript title: Effects of ultrasonication on increased germination and improved seedling growth of aged grass seeds of tall fescue and Russian wildrye

Authors: Juan Liu[1], Quanzhen Wang[1], Đura Karagić[2], Xv Liu[1], Jian Cui[3], Jing Gui[1], Muyu Gu[1], Wei Gao[1]

[1]College of Animal Sci. and Techn., Northwest A&F University, Yangling 712100, Shaanxi Province, China. [2]Institute of Field and Vegetable Crops, Forage Crops Department, Maksima Gorkog 30, 21000 Novi Sad, Serbia. [3]College of Life Science, Northwest A&F University, Yangling 712100, Shaanxi Province, China,

Number of pages: 4.

Number of Supplementary Figures: 1. Number of Supplementary Tables: 4.

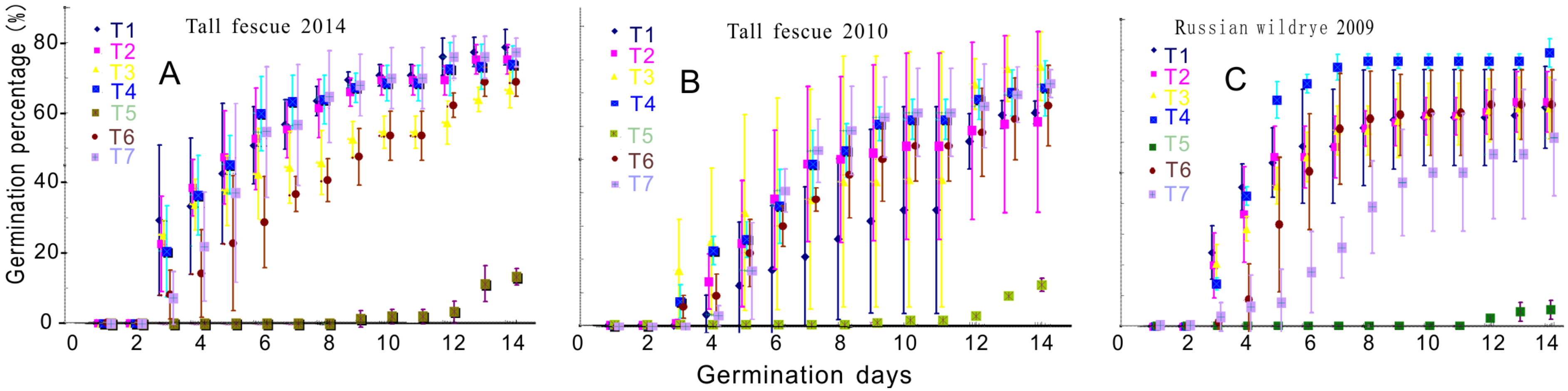

Figure S1. Germination percentage in the treatments for the tall fescue (2014 (A) and 2010 (B)) and the Russian wild rye 2009 (C) seeds with Average ± Standard Deviation. T1 through T6 are the treatments, and T7 is the control.

Table S1 Pearson correlation coefficients for germination percentage (GP), germination index (GI), seed vigour index (SVi), shoot length, root length, MDA content, and activity of SOD and POD.

0	GI	SVi	Shoot length	Root length	MDA	SOD	POD
GP	0.217***	0.064	0.819***	0.763***	-0.138*	0.097	-0.042
GI	1.000	0.336***	0.292***	0.392***	-0.062	0.043	0.235***
SVi		1.000	-0.026	-0.007	0.065	-0.083	-0.122*
Shoot length			1.000	0.971***	-0.280***	0.185**	0.110
Root length				1.000	-0.259***	0.179**	0.209**
MDA					1.000	-0.166*	0.068
SOD						1.000	0.181**

F-values are presented with significant differences: *P < 0.05, **P < 0.01, and ***P < 0.0001.

Table S2. The constants *a*, *b* and *c* of the logistic models for different treatments T1 to T7 in tall fescue 2014, 2010 and Russian wild rye 2009.

		a	b	c
Tall fescue 2014	T1	2.263	0.605	75.36
	T2	2.463	0.752	70.035
	T3	1.962	0.578	59.327
	T4	3.01	0.949	70
	T5	8.978	0.820	13.841
	T6	2.46	0.397	72
	T7	3.541	0.868	73.22
Tall fescue 2010	T1	3.58	0.584	62
	T2	4.268	0.977	57.42
	T3	2.567	0.811	70.847
	T4	3.042	0.631	68.956
	T5	11.694	1.068	10.657
	T6	3.3	0.62	61.754
	T7	6.02	1.248	66.472
Russian wild rye 2009	T1	3.331	1.28	66.353
	T2	3.175	1.066	68.5
	T3	3.022	0.916	69.66
	T4	5.051	1.677	86.478
	T5	38.436	3.426	5.469
	T6	5.262	1.255	71.151
	T7	4.108	0.66	58.941

Table S3 Seeds of grasses harvested in different years in the nested experiment.

Harvested years	Tall fescue	Group No.	Russian wild rye	Group No.
Level 1	2014	T1	2009	R1
Level 2	2010	T2	2006	R2
Level 3	2006	T3	2003	R3

Table S4 The L_9 (3^4) matrix with associated factors.

Treatments	Sonication time (min)	Sonication temperature (℃)	Output power (W)	Seed soaking time (h)
1	1 (15)	1 (25)	3 (500)	2 (5)
2	2 (35)	1	1 (200)	1 (1)
3	3 (55)	1	2 (350)	3 (9)
4	1	2 (45)	2	1
5	2	2	3	3
6	3	2	1	2
7	1	3 (65)	1	3
8	2	3	2	2
9	3	3	3	1
Control	0	---	0	0

Table S5 Basic statistics for the combined effects of sonication temperature, sonication time and ultrasound output power on the germination (%) of tall fescue (2014 and 2010) and Russian wild rye (2009) seeds. *: Average± Standard Deviation

Treatments	Tall fescue 2014	Tall fescue 2010	Russian wild rye 2009
1	78.7 ±5.0*	64.0±3.5	71.3±13.3
2	75.3±4.2	61.3±27.2	73.3±9.9
3	66.7±5.0	79.0±9.5	71.3±10.1
4	74.0±5.3	71.3±8.3	89.3±4.6
5	13.3±2.3	12.0±2.0	5.3±3.1
6	68.7±4.2	66.0±12.0	72.7±11.4
Control	77.3±4.2	72.7±4.2	61.3±19.0
